# Exploring the Determinants of Tobacco Usage Among Adolescents: A Cross-Sectional Study in Western Maharashtra

**DOI:** 10.7759/cureus.66755

**Published:** 2024-08-13

**Authors:** Shweta Gangurde, Shubham Shivale, Hetal Rathod, Prerna Verma, Divya Madamanchi, Anil Mahajan, Pratap B Kaushik, Johnson S., Sai Mahesh Vajjala

**Affiliations:** 1 Community Medicine, Dr. D. Y. Patil Medical College, Hospital and Research Centre, Dr. D. Y. Patil Vidyapeeth, Pune (Deemed to be University), Pune, IND

**Keywords:** socio-demographic factors, family influence, peer pressure, smoking, smokeless tobacco, adolescents, tobacco use

## Abstract

Introduction

The widespread use of tobacco is a major global health threat, causing significant morbidity and mortality. The World Health Organization (WHO) estimates that annually, millions of people die prematurely due to tobacco use, with this number projected to increase significantly in the future. Developing countries, including India, bear a substantial burden of tobacco-related deaths, often beginning during adolescence. This study estimated the prevalence of tobacco use among adolescents in the 13-19 years age group, identified the types of tobacco products used, explored the reasons for initiation, and examined the influence of family, peers, and socio-demographic factors on tobacco use.

Methods

A community-based cross-sectional study was conducted from August 2022 to June 2024 in a medical college's urban and rural field practice areas in Pune district, Maharashtra. A total of 310 adolescents were surveyed using convenience sampling. Data were collected using a pre-designed, pre-tested questionnaire, and statistical analysis was performed using Jamovi software version 2.3.28. Chi-square and Fisher's exact tests were applied to assess associations between variables.

Results

Out of 310 participants, 94 (30.32%, 95% CI: 25.25%-35.77%) reported using tobacco. Among these 94 participants, 82 (87.23%) used smokeless tobacco, 19 (20.21%) used the smoked form of tobacco, and seven (7.45%) used both forms. The mean age of initiation was 15.94 years for smoking and 15.59 years for smokeless tobacco. The data indicate a higher percentage of tobacco use among females (50%) compared to males (24.8%). However, in terms of absolute numbers, more males (60) than females (34) reported using tobacco due to the larger number of males in the study sample. The most common reasons for initiation included curiosity (43.74%) and peer pressure (53.99%). Specifically, 49 individuals (42.6%) aged 13-15 were using tobacco, compared to 23 individuals (30.3%) aged 16-17, and only 22 individuals (18.5%) aged 18-19. Participants from urban areas reported higher tobacco use (48%) compared to those from rural areas (46.7%). Among those from joint families, about 24 (31.6%) reported tobacco use, while in nuclear families, it was slightly lower at about 70 (29.9%).

Conclusion

Factors such as peer pressure and curiosity played significant roles in the initiation of tobacco use, with more than half of the participants citing peer influence as the primary reason for starting. This study revealed differences in tobacco usage patterns across age groups, with younger participants showing higher usage rates. The findings highlight the need for targeted interventions, such as health education and anti-tobacco media campaigns, to reduce tobacco use among adolescents.

## Introduction

The widespread use of tobacco poses a significant threat to global health, being a major avoidable cause of illness and disability across the world. According to World Health Organization (WHO) estimates, every year, 5 million individuals die prematurely due to tobacco and tobacco-related product usage, and this number is expected to double to 10 million by 2030 [1]. Most of these deaths, around 7 million, are expected to occur in developing countries. India is anticipated to experience the most rapid increase in tobacco-related deaths, many of which will happen during the productive adult years as a result of addiction acquired in youth [1].

India faces a particularly intricate tobacco issue, surpassing that of most other nations, resulting in a significant burden of tobacco-related illnesses and fatalities. According to the National Survey on Drug Use and Health, tobacco use typically begins during childhood and adolescence [2]. The Global Youth Tobacco Survey (GYTS) provides data on tobacco use among adolescents aged 13-15 years. In India, the GYTS-4 (2019) reported that 8.5% of students aged 13-15 years use some form of tobacco, with a higher prevalence among boys (9.6%) compared to girls (7.4%) [3]. According to the National Family Health Survey (NFHS Phase 5), which was conducted in 2019 and 2021, tobacco consumption in India among individuals aged 15 and older was 38% for men and 9% for women. In Maharashtra state, among individuals aged 15 and older, the prevalence of tobacco use was 33.8% for males and 11% for females [4].

Addiction refers to a strong and lasting desire to engage in specific behaviours [5]. The powerful attraction to tobacco is driven by the presence of nicotine, a highly potent psychoactive substance found in it [6,7]. This substance is responsible for making tobacco the second leading cause of death worldwide [8,9].

This study is essential due to the significant global health threat posed by tobacco, a major preventable cause of illness and disability. In India, tobacco use begins early and contributes to a high burden of disease and death, with addiction driven by nicotine. The purpose of this study was to find out the prevalence of tobacco use among adolescents aged 13 to 19 years, examining the variety of tobacco products they use, the reasons for starting, and the factors influencing their tobacco habits. It also investigated the role of family conversations, the effect of peer influence, and demographic characteristics on their tobacco use.

## Materials and methods

This study employed a community-based cross-sectional design and was conducted in urban and rural field practice areas of a medical college in Pune district, Maharashtra, from August 2022 to June 2024. The research focused on adolescents from three randomly selected villages. All villages within the field practice area were listed, and three villages were chosen using simple random sampling. Additionally, an Urban Health Training Centre, located in a slum area within the field practice area of the medical college in Pune, was included in the study.

Sample size and sampling method

Sample Size

According to a study by Nazir et al., the prevalence of tobacco use was 19.33% [10]. Considering the above prevalence, an acceptable difference of 4.5%, a 95% confidence interval, 80% power, and a non-response rate of 4%, the minimum sample size was calculated to be 309. The software used was WinPepi (PEPI-for-Windows), version 11.38. However, 310 participants were included in the study.

Participants aged 13-19 who provided prior consent and assent and did not have any neuropsychiatric illnesses were included in the study. Conversely, individuals who were critically ill, had chronic diseases, or did not give consent were excluded.

Sampling Method

A house-to-house survey was conducted in both urban and rural villages within the medical college's field practice area. Eligible participants aged 13 to 19 years were interviewed using a pretested, prevalidated structured questionnaire. For minors (under 18 years), both parental consent and assent (the minor's agreement to participate) were obtained, while participants aged 18 years and older provided their own informed consent. In cases where households were closed during the initial visit, follow-up visits were made the next day. If the household remained closed, an alternate household was approached. In total, 160 participants were interviewed in the urban slum field practice area and 150 participants were interviewed in the rural field practice area, with 50 subjects from each of the three selected villages.

Ethical approval

The Institutional Ethics Committee approved the study protocol via reference number IESC/PGS/2022/207. The participants were assured of the complete confidentiality of their personal information. It was explained to them that their participation was voluntary, and they could opt out of the study at any point.

Data collection method

A pre-designed and pre-tested questionnaire, comprising approximately 40 questions, was utilized for data collection. Individual interviews were conducted with each participant in a private setting. The questionnaire was administered in both Marathi and Hindi languages.

The operational definitions for current and past smokers were set as follows: Current users - individuals who have been using tobacco products daily for at least the past month. Past users - individuals who haven't used tobacco in the last month but used it earlier.

Statistical analysis

Descriptive statistics, including proportions, means, standard deviations, medians, and interquartile ranges, were computed as required to examine the distribution of study variables. The normality of the data was checked by the Shapiro-Wilk test. Frequency distributions of study participants, based on factors such as age, gender, family type, residence, and friends' habits, were analysed. The association between tobacco use and these variables was tested using the Chi-square test with a significance level of p < 0.05. Fisher's exact test was utilized where appropriate. Statistical analysis was conducted using Jamovi version 2.3.28.

## Results

The study involved 310 participants. Most of them were boys (78%). The participants involved in the study were selected from both rural (48.4%) and urban (51.6%) areas of the field practice area in Pune. Three age groups were created: 13-15 years old, 16-17 years old, and 18-19 years old. Most of them had completed eighth grade (85.5%). The families of 80% of the participants earned ₹15,000 or more every month. The majority of participants were from nuclear families (75.5%), while 24.5% were from joint families. Many of the heads of families worked in services (34.84%) or as farmers (25.71%). Predominantly, the occupation of the parents of the participants was service (employed) (34.84%), followed by farmers (25.71%).

Out of 310 adolescents surveyed, 94 (30.32%) reported using tobacco. The confidence intervals indicate the probable range of the true proportions of tobacco users in each category (Table 1). Among these 94 users, 82 (87.23%) used chewable tobacco. A smaller group of 19 (20.21%) participants reported smoking tobacco. Only seven individuals (7.45%) indicated that they used both chewing and smoking forms of tobacco (Table 2).

**Table 1:** Prevalence of tobacco use

Total participants (N = 310)	N (%)	95% CI
Participants using tobacco	94 (30.32%)	25.25-35.77
Participants not using tobacco	216 (69.67)	64.23-74.75

**Table 2:** Prevalence of tobacco use in various forms The combined percentage of smokeless tobacco users, smoking tobacco users, and individuals using both forms exceeds 100% because the categories for both forms include individuals who use both smokeless and smoking tobacco, resulting in a total greater than 100%.

Form of tobacco	Number of users	Total tobacco users in either form	Percentage
Smokeless tobacco users	82	94	87.23%
Smoking form of tobacco users	19	94	20.21%
Both the forms	7	94	7.45%

In this study, all tobacco smokers were boys, accounting for 100% of the male smokers. None of the girls reported smoking. However, among those who used chewing tobacco, most were men, about 81.70%, while 18.29% were women. Similarly, for those who used both chewing and smoking tobacco, most were men, comprising approximately 71.42%, while about 28.57% were women. This indicates that tobacco smoking is more prevalent among men than women. However, both men and women use chewing tobacco, with a higher usage rate among men.

**Figure 1:**
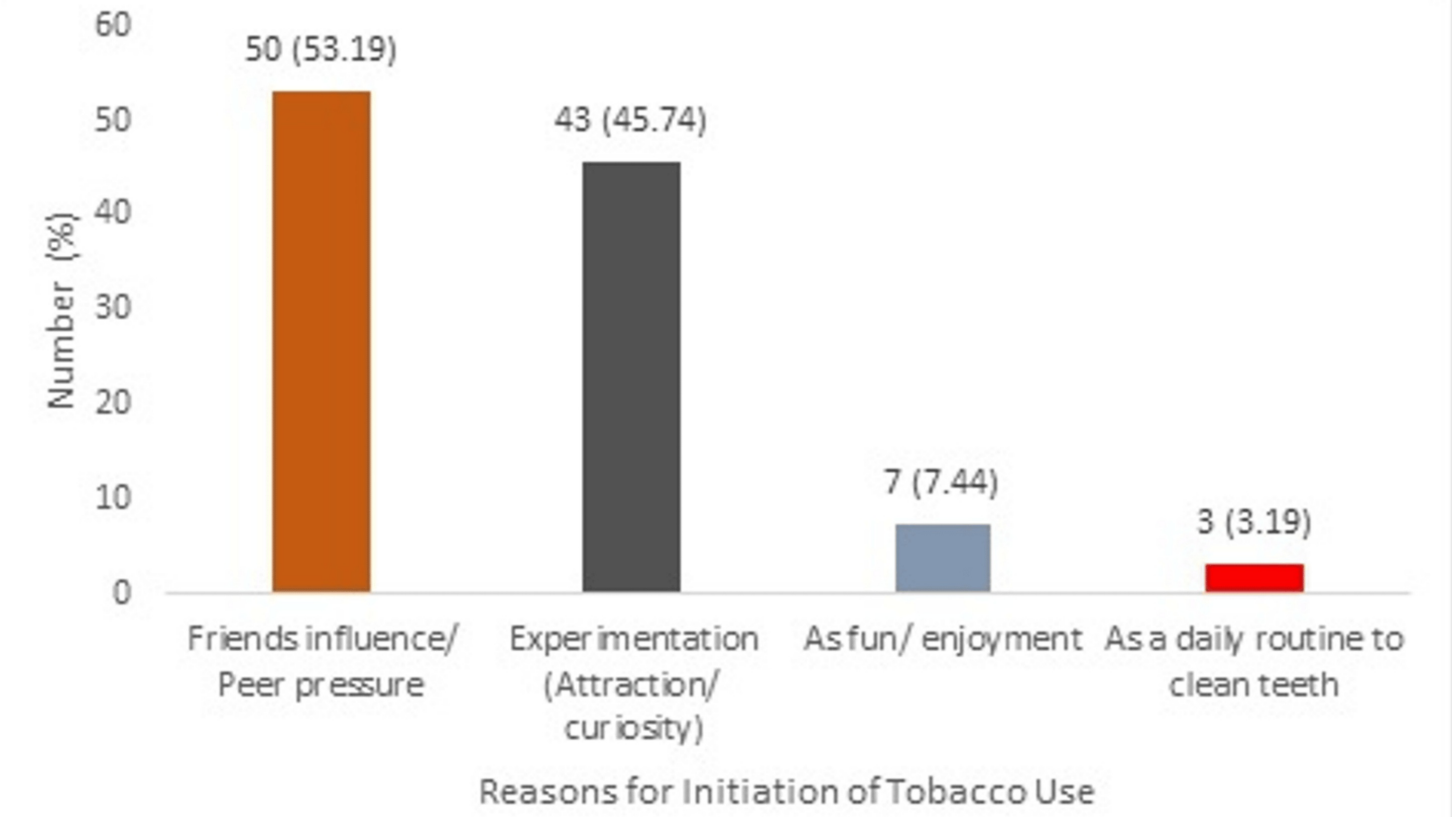
Reasons for initiation of tobacco use among adolescents (N = 94) The percentages for tobacco initiation reasons didn’t total 100% because respondents chose multiple reasons. In surveys permitting multiple answers, this can cause the total percentage to be more or less than 100%.

The reasons for the initiation of tobacco use among the study subjects were different. The most common reason was friends' influence or peer pressure, as reported by 50 participants (53.19%). Experimentation due to attraction or curiosity was cited by 43 participants (45.74%). A smaller number of participants, seven (7.44%), started using tobacco for fun or enjoyment. Only three participants (3.19%) of the 94 users reported using tobacco as a daily routine to clean their teeth (Figure 1).

The mean age (SD) of initiation of tobacco in smoke form and smokeless form was 15.94 (1.13) years and 15.59 (1.26) years, respectively. Among the 94 participants, Mawa (a mix of tobacco with lime, areca nut, and flavours) was consumed by 66 (70.21%) participants. Gutkha is also common, with 61 (64.89%) people using it. Pan masala mixed with tobacco is liked by around 26 (27.65%) participants. Mishri was used by three (3.19%). This shows that people in this group have different preferences when it comes to tobacco products. Additionally, 19 participants (20.21%) smoke cigarettes.

The data on tobacco use among different age groups shows a significant trend, where younger adolescents are more likely to use tobacco than older ones (Table 3).

**Table 3:** Sociodemographic characteristics and tobacco use Data are presented as N (%), with p-values determined by the χ² test. A p-value of <0.05 is considered statistically significant. *OR = 0.508; **OR = 0.305

Characteristics		Tobacco users (N = 94)		Non-tobacco users (N = 216)		Total		Test, p-value
Study participants (total = 310)		N	%	N	%	N	%	
Age group (in years)	13-15	49	42.6	66	57.4	115	37	χ² for trend = 16.05, p < 0.001
	16-17*	23	30.3	53	69.7	76	24.51	
	18-19**	22	18.5	97	81.5	119	38.38	
Residence and habit of tobacco use	Rural	46	30.7	104	69.3	150	48.38	χ^2^ = 0.016, p-value = 0.898
	Urban	48	30	112	70	160	51.61	
Gender-wise distribution of tobacco use	Females	34	50	34	50	68	21.93	χ^2^ =16, p < 0.001
	Males	60	24.8	182	75.2	242	78.06	
Education and tobacco habit	Less than secondary (<10th)	91	41.7	127	58.3	218	70.32	χ^2^ = 45.3, p = 0.001
	Higher secondary (11th-12th)	3	3.3	89	96.7	92	29.6	

Specifically, 42.6% of individuals aged 13-15 years use tobacco, compared to 30.3% of those aged 16-17, and only 18.5% of those aged 18-19. This decline in tobacco use with age is statistically significant, as indicated by the Chi-square (for trend) test result (χ² = 16.05, p < 0.001).

Among those whose friends use tobacco, 24 participants (31.2%) were also tobacco users. Likewise, among those whose friends don't use tobacco and those unsure about their friends' tobacco use, 51 participants (31.1%) and 19 participants (27.5%) used tobacco, respectively. Statistical analysis indicates no significant association between an individual's tobacco use and their friends' tobacco use status, with a p-value of 0.849 and a Chi-square test result of 0.326 (Table 4).

**Table 4:** Determinants of tobacco use Data are presented as N (%), with p-values determined by the χ² test. A p-value of <0.05 is considered statistically significant.

Characteristics	Tobacco users N (%)	Non-tobacco users N (%)	Total N (%)	Test, p-value
Tobacco use and its relation with type of family				
Joint	24 (31.6)	52 (68.4)	76 (24.51)	χ^2^ = 0.07, p-value = 0.78
Nuclear	70 (29.9)	164 (70.11)	234 (75.48)	
Friends using tobacco in some form				
Yes	24 (31.2)	53 (68.8)	77 (24.83)	χ^2^ = 0.32, p-value = 0.849
No	51 (31.1)	113 (68.9)	164 (52.90)	
Don’t know	19 (27.5)	50 (72.5)	69 (22.25)	
Family discussion about health effects and tobacco use				
Yes	83 (29.1)	202 (70.9)	285 (91.93)	χ^2^ = 2.4, p-value = 0.121
No	11 (44)	14 (56)	25 (8.06)	
Family members using tobacco				
Yes	34 (28.6)	85 (71.4)	119 (38.38)	χ^2^ = 0.28, p-value = 0.596
No	60 (31.4)	131 (68.6)	191 (61.61)	
Taught by teachers ever about tobacco effects				
Yes	84 (29.4)	202 (70.6)	286 (92.25)	χ^2^ = 1.58, p-value = 0.208
No	10 (41.7)	14 (58.3)	24 (7.74)	

Among participants whose family members were using tobacco, 28.6% reported using tobacco themselves, while among those without family members using tobacco, the percentage was slightly higher at 31.4%. The Chi-square test result (χ² = 0.280, p = 0.596) indicates no significant association between family members' tobacco use and individual tobacco use (Table 4).

Among those who discuss tobacco use with their family, 29.1% were tobacco users, compared to 44% among those who don't have these discussions. However, statistical analysis reveals no significant association between family discussions about health effects and tobacco use, with a p-value of 0.121 (Table 4). Among individuals who received education from teachers about the health effects of tobacco, 29.4% were tobacco users. In contrast, 41.7% of those who did not receive this instruction were using tobacco. The statistical analysis indicates no significant association between being taught about tobacco effects and tobacco use, as evidenced by a Chi-square test result of 1.58, with a p-value of 0.208 (Table 4).

## Discussion

Tobacco use poses a long-term health risk and is associated with non-communicable conditions such as chronic respiratory diseases, cancer, diabetes, heart disease, stroke, and premature mortality. The WHO predicts that India will experience the most rapid increase in tobacco-related deaths during the initial two decades of the 21st century. Many of these deaths are the result of early-onset addiction, implying that many occur during the productive years of life [11].

As in this study, the rate of tobacco use in multiple forms among adolescents was 30.32%. Among females, 50% of participants reported using tobacco, evenly divided with those reporting no use. In contrast, among males, a lower percentage (24.8%) reported no use. This variance between both genders was statistically significant (p-value ≤ 0.0001). In contrast, according to the findings of a report on tobacco control in India, the prevalence of current smoking among adolescents stood at 8.3% nationwide. This percentage varied across different regions, ranging from 2.2% in Himachal Pradesh to as high as 34.5% in Mizoram, as indicated by various research studies [11].

The age at which individuals started using tobacco was 22.62 years, which was consistent with findings from research conducted by Joshi et al. among 2,513 individuals from urban Jamnagar [12]. In contrast, a study by Ayyappa et al. among 590 participants aged above 15 years reported that the age at initiation was below 18 years [13]. According to a study by Raval et al., the age of initiation of tobacco use was below 10 years [14]. Our study found that the mean age (SD) of initiation of tobacco in smoke form and smokeless form was 15.94 (1.13) years and 15.59 (1.26) years, respectively. Tobacco use is highest among individuals aged 13-15 years, with 42.6% reporting being users, compared to 30.3% among 16-17-year-olds and 18.5% among those aged 18-19 years.

Joshi et al. noted that the prevalence of tobacco chewing rises with advancing age [12]. According to this study, the prevalence of tobacco chewing was approximately 10% among the 13-17 age group, increasing to 51.3% among those aged 17-19 years. A study by Pednekar and Gupta highlighted that tobacco usage among both parents and peers significantly impacts the likelihood of adolescents being current smokers [15]. Similar to this study, the data highlight a notable prevalence of smokeless tobacco use among participants and their friends, with about 24.83% of participants and 26.45% of their friends reporting using it. This indicates both individual usage and significant peer influence. Addressing this dual aspect of personal use and social influence is vital for effective tobacco prevention and cessation strategies. People whose friends don't use tobacco are also less likely to use it themselves (p < 0.001).

There was a notable correlation between the habit of tobacco use among friends and the current use among the participants in the study. Several influential factors identified in our study, as well as in previous research, have demonstrated a significant correlation with tobacco use among adolescents. These factors include a positive history of tobacco use among parents and peers, insufficient awareness regarding the harmful effects of tobacco, and a favourable attitude towards individuals who use tobacco [15].

In the study, it was discovered that in the last 30 days, 84.5% of students faced direct exposure to anti-smoking messages through media, such as those seen on television, heard on the radio, displayed on posters, published in newspapers, or featured in movies. Madan Kumar et al. found that almost everyone saw cigarette ads on TV, and about half saw them on billboards outdoors (45.7%), in newspapers (65.3%), and at gatherings (67.4%) [16]. Integrating health education into the curriculum and informing students about the negative consequences of smoking can play a vital role in delaying or reducing smoking rates among students. School-based educational programmes and support for quitting can improve outcomes related to tobacco use. Additionally, training health professionals and teachers can help ensure these interventions are effectively implemented [17].

In terms of tobacco consumption patterns, 94 (30.32%) of the participants were using tobacco in either form. Among those 94 users, 82 (87.23%) were using smokeless tobacco, whereas 19 (20.21%) were using smoking tobacco. Seven users (7.45%) were reported to be using both forms. Similarly, a study by Surekha et al. in rural Wardha found that 41.35% of adolescents used smokeless tobacco, while 5.48% were smokers [18]. Another study by Dongre et al. among rural Wardha adolescents reported that 39% used smokeless tobacco, with only 1.29% smoking bidi or cigarettes in the past month [19].

The study pointed out that curiosity about experiments and peer pressure were the major reasons why people began consuming tobacco. These findings are similar to the studies conducted by Ayyappa et al. [13]. A study done in Chennai by Chockalingam et al. showed that, in comparison to semi-urban (20.9%) and urban (19.4%) areas, the overall prevalence of tobacco usage was much higher in rural areas (23.7%), with a p-value of less than 0.001 [20]. In contrast, our study found that 48 (30%) of participants residing in urban areas were consuming tobacco, indicating a higher tendency for study subjects from urban areas to use tobacco.

The Cigarettes and Other Tobacco Products (Prohibition of Advertisement and Regulation of Trade and Commerce, Production, Supply, and Distribution) Act of 2003 prohibits tobacco advertising and smoking. Strict enforcement of this law is essential to reducing smoking rates among both youths and adults. Worldwide, over a third of the population regularly faces the harmful effects of smoking, resulting in approximately 600,000 deaths annually and contributing to around 1% of the global disease burden [21].

Limitations

The study has several limitations. First, the significant gender imbalance, with 78.1% male participants, may introduce bias and limit the applicability of the findings to a more balanced adolescent population. Second, focusing exclusively on specific urban and rural areas within the Pune district may restrict the generalizability of the results to broader regional or national contexts. Third, the reliance on self-reported data could lead to underreporting of tobacco use due to social desirability bias, potentially skewing prevalence estimates. Additionally, the cross-sectional design of the study limits the ability to establish causal relationships between identified factors and tobacco use patterns over time. Addressing these limitations in future research could improve the comprehensiveness and applicability of findings, aiding in the development of more effective tobacco control policies and interventions.

## Conclusions

This study included a substantial number of participants and found that a notable portion reported using tobacco. Among these, a significant number used smokeless tobacco, while a smaller group used smoking tobacco, with only a few using both types. Smoking tobacco was more common among males, whereas the use of smokeless tobacco was more evenly distributed across genders. Peer pressure and curiosity were identified as major factors in initiating tobacco use, with many participants attributing their start to peer influence. The study also revealed variations in tobacco use patterns by age, with younger participants showing higher rates. Family structure played a role, as those from joint families reported higher tobacco use compared to those from nuclear families. Overall, the study highlights the intricate relationship between socio-demographic factors and personal influences in shaping tobacco use among adolescents.
